# Special Issue “Molecular Engineering for Electrochemical Power Sources”

**DOI:** 10.3390/molecules21111524

**Published:** 2016-11-12

**Authors:** Sergei Manzhos

**Affiliations:** Department of Mechanical Engineering, National University of Singapore, Block EA #07-08, 9 Engineering Drive 1, Singapore 1175761, Singapore; mpemanzh@nus.edu.sg; Tel.: +65-6516-4605

Electrochemical energy conversion and storage technologies are expected to play an increasingly prominent role in the production and consumption of electricity in the coming decades. They hold the promise of economic solar-to-electricity or solar-to-fuel energy conversion, high response rate grid storage, and are enabling the spread of all-electric vehicles. These technologies include metal ion batteries, dye sensitized and organic solar cells, fuel cells and redox flow batteries. While their respective research fields have mostly been developing in parallel, these technologies have to address similar issues of *molecular* engineering, e.g., band gap engineering, redox level tuning, optimization of electron transfer rates; these concerns crop up in electrolyte design for Li or post-Li electrochemical batteries, organic electrode materials for emerging organic batteries, chromophore and redox shuttle molecules for dye-sensitized cells and redox flow batteries, among others. This Special Issue aims to foster cross-fertilization of ideas in molecular engineering coming from these different fields. 

This short Special Issue features both experimental and theoretical works which cover the topics of design of molecular structure, optimization of molecule–semiconductor interfaces, and optimization of electron transfer rates. These works consider molecular engineering from the perspective of electrochemical energy conversion (specifically, molecular chromophore based solar cells and water splitting) and storage (specifically, redox flow batteries). The review by Pan and Wang [1] of redox species used in redox flow batteries showcases how molecular engineering can help address the biggest challenge that this electrochemical energy storage technology faces, namely, low energy density. The review highlights recent developments in redox species, specifically, how the design of novel, molecular redox shuttles for non-aqueous redox flow batteries allows for increased voltage and energy density. 

The requirements of suitable redox potentials and of minimal thermal losses during the electron transfer process are common to redox flow batteries and photoelectrochemical cells. Indeed, some of the most promising types of redox shuttles are common to the field of redox flow batteries and dye-sensitized cells (e.g. transition metal-based bi-pyridines). These two fields can and do benefit from an exchange of ideas. Several papers in this Special Issue address issues facing photoelectrochemical cells. The papers by Zhang et al. [2] and Raynor et al. [3] are concerned with chromophore design, respectively, metal–organic [2] and organic [3]. The work by Zhang et al. [2] presents a combined experimental-computational study of a so-called “red sensitizer”, an osmium ter-pyridine. Unlike some widely researched Ru-based analogues, Os based dyes promise panchromatic absorption in the full visible range and hold promise for use in efficient dye-sensitized solar cells. Raynor et al. [3] design a new all-organic molecular chromophore for bulk heterojunction solar cells; they use, for the first time, a thiophene unit in conjunction with a 1,4-phenylenediacetonitrile acceptor moiety to extend the π-conjugation in a D–A–D motif and thereby achieve a significant improvement of the photocurrent.

Two articles in this issue consider properties of interfaces providing the functionality of a photoelectrochemical cell. The work by Manzhos et al. [4] considers, in a computational study using density functional tight-binding, different absorption modes of a carboxylate-terminated molecule on low-energy surfaces of several phases of titania. Strong dye-surface binding is critical to achieve both efficient electron transfer and cell stability in dye-sensitized solar cells; the work of Manzhos et al. allows comparing, at the same level of approximation, adsorption on different surfaces including amorphous, where they predict that very strong adsorption can be achieved. The paper by Gomez et al. [5] presents a model of the photoelectron transfer process between alizarin and titania using an explicitly time-dependent one-electron time-dependent density functional theory approach and a set of home-made analysis tools. Their work contributes to the still relatively scarce literature (and relatively scarce choice of methods and software) explicitly describing the electron injection process. 

Finally, the work of Yatom and Toroker [6] addresses doping as a strategy of improving the efficiency of photoelectrochemical cells used for water splitting. Doping is a popular strategy to improve visible absorption and conductance and has been used on wide-gap semiconductors used in water splitting and photocatalysis in general, and in solar cells. The improvement of these two properties, however, does not necessarily result in improved energy conversion efficiency. In the paper of Yatom and Toroker, the effects of *n*-doping on properties determining photocatalytic performance are analyzed from the ab initio perspective, on the example of Nb-doped Fe_2_O_3_. They identify correlations between the charge of the dopant, the charge on the surface of the Fe_2_O_3_ material, and the overpotential required for water oxidation. This information can be useful in the design of improved doped photocatalysts.

Electrochemical power sources (EPS) will continue to be of intense research interest for the foreseeable future. Molecular engineering for different EPS, deals with many similar issues, both in computational and experimental design. This Special Issue has attempted to bring several works addressing these issues for different applications under the same roof, and we hope it will provide a useful perspective for researchers in working on any EPS technology.
